# Risk factor analysis for progressive spinal deformity after resection of intracanal tumors─ a retrospective study of 272 cases

**DOI:** 10.1186/s12883-019-1594-x

**Published:** 2020-01-23

**Authors:** Pangbo Wang, Kang Ma, Tunan Chen, Xingsen Xue, Dada Ma, Shi Wang, Xin Chen, Hui Meng, Gaoyu Cui, Boyuan Gao, Jiangkai Lin, Hua Feng, Weihua Chu

**Affiliations:** Department of Neurosurgery, Southwest Hospital, Third Military Medical University (Army Medical University), No. 29, Gaotanyanzheng Street, Shapingba District, Chongqing, 400038 China

**Keywords:** Intracanal tumors, Progressive spinal deformity, Risk factors

## Abstract

**Background:**

Progressive spinal deformity has become a well-recognized complication of intracanal tumors resection. However, the factors affecting post-operative spinal stability remain to be further research. Here, we described the current largest series of risk factors analysis for progressive spinal deformity following resection of intracanal tumors.

**Methods:**

We retrospectively analyzed the medical records of the patients with resection of intracanal tumors between January 2009 and December 2018. All patients who underwent resection of intracanal tumors performed regular postoperative follow-up were identified and included in the study. Clinical, radiological, surgical, histopathological, and follow-up data were collected. The incidence of postoperative progressive kyphosis or scoliosis was calculated. The statistical relationship between postoperative progressive spinal deformity and radiographic, clinical, and surgical variables was assessed by using univariate tests and multivariate logistic regression analysis.

**Results:**

Two hundred seventy-two patients (mean age 42.56 ± 16.18 years) with median preoperative modified McCormick score of 3 met the inclusion criteria. Among them, 7(2.6%)patients were found to have spinal deformity preoperatively, and the extent of spinal deformity in these 7 patients deteriorated after surgery. 36 (13.2%) were new cases of postoperative progressive deformity. The mean duration of follow-up was 21.8 months (median 14 months, range 6–114 months). In subsequent multivariate logistic regression analysis, age ≤ 18 years (*p* = 0.027), vertebral levels of tumor involvement (*p* = 0.019) and preoperative spinal deformity(*p* = 0.008) was the independent risk factors (*p* < 0.05), increasing the odds of postoperative progressive spinal deformity by 3.94-, 0.69- and 27.11-fold, respectively.

**Conclusions:**

The incidence of postoperative progressive spinal deformity was 15.8%, mostly in these patients who had younger age (≤18 years), tumors involved in multiple segments and preoperative spinal deformity. The risk factors of postoperative progressive spinal deformity warrants serious reconsideration that when performing resection of spinal cord tumors in these patients with such risk factors, the surgeons should consider conducting follow-ups more closely, and when patients suffering from severe symptoms or gradually increased spinal deformity, surgical spinal fusion may be a more suitable choice to reduce the risk of reoperation and improve the prognosis of patients.

## Background

Primary spinal cord tumors are rare, with an incidence of 0.76 per 100,000 in the United States [1]. Advances in intraoperative neuroelectrophysiological monitoring and microsurgical techniques have contributed to the success rate of spinal cord tumors (SCTs) resection, which increases long-term survival and improves the quality of life of patients with spinal cord tumors [2, 3]. However, with improved survival and longer follow-up times, patients often develop progressive spinal deformity postoperatively in the years after surgery, and which cause postoperative pain, reoperation and neurologic compromise in patients. It is reported that incidence of spinal deformity following intradural spinal tumor resection up to 10% in adults and rates ranging from 16 to 100% in pediatric patients [4–7]. Some reports suggested that laminoplasty resulted in less spinal deformity for the partial restoration of the posterior tension band [4]. But recent studies indicated that the incidence of postoperative spinal deformity associated with laminoplasty is close to laminectomy [8–10]. In fact, the study about risk factors for progressive spinal deformity after spinal cord tumors resection using the laminoplasty has been conducted [11]. However, limited by small samples and absence of multivariate analysis in previous studies, the convincing risk factors are still unclear. We undertook this larger retrospective study to furthermore determine the risk factors which may result in the higher incidence of progressive spinal deformity after surgical treatment of intracanal tumors.

## Methods

**Fig. 1:**
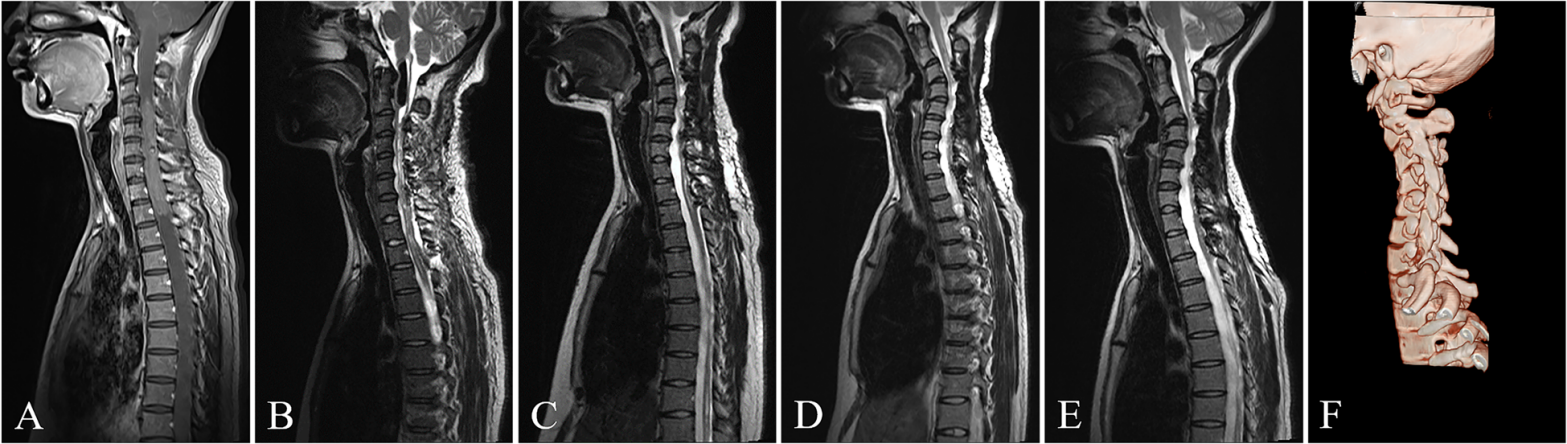
The T2-weighted MR images shown was a 30-year-old woman who underwent resection of the ependymoma spanning from C3 to T2. **a** Preoperative MR images showed a huge tumor and the cervical spine had lost its normal curvature. **b** Postoperative MR images during postoperative hospitalization showed complete resection of spinal cord tumor and almost no change in cervical curvature. **c** The follow-up MR image showed progressive cervical kyphosis 6 months after the operation. **d**, **e** The follow-up MR image showed deterioration of progressive cervical kyphosis 13 months and 24 months after the operation, respectively. **f** The CT three-dimensional reconstruction image showed the cervical kyphosis 24 months after the operation. At that time, the patient complained that her left upper limb was numb

This research was approved by the Ethics Board of Southwest Hospital of Army Military Medical University in China. The medical records of all patients underwent intracanal tumors resection between January 2009 and December 2018 at Chongqing Southwest Hospital were retrospectively analyzed. All patients who underwent resection of intracanal tumors performed regular postoperative follow-up were identified and included in the study. Exclusion criteria included: (1) previous resection at the same location; (2) ever underwent tumor resection with concurrent fusion. 272 patients were identified and included in the study. All clinical and radiological variables of the patients were recorded. All the patients underwent preoperative and postoperative imaging assessments (plain lateral radiographs and MRI) and were followed up at 3-, 6-, 12-, 18-, and 24-month after surgery to assess the presence of tumor recurrence and spinal deformity. The key endpoint of this study was the occurrence of progressive spinal deformity (Fig. 1). Progressive spinal deformity was defined as the progression of kyphotic or scoliotic curves by at least 10° on 2 or more consecutive radiographs. Preoperative coronal Cobb angles > 10°, less of cervical/lumbar lordosis and kyphosis of the cervical/thoracic/lumbar spine before SCTs resection were classified as preoperative spinal deformity. Progressive spinal deformity was first treated conservatively with prolonged bracing for another 3 to 6 months. The fusion surgery would be considered if it continued to develop or had symptoms. Neurological examinations of patients preoperatively, at hospital discharge, and regular follow-up were recorded. Functional status was evaluated according to the modified McCormick scale (MMS) both preoperatively and at the last follow-up [12].

In most cases, gross-total resection of the tumor, defined as excision of ≥95% of the tumor or absence of residual enhancement on postoperative MRI, was performed. In other cases, subtotal resection (80–95% resection) were made when the tumors invaded some more important tissues. Meanwhile, immediate postoperative MRI may detect retained fragment. Partial resection (removal of < 80% of the tumor) only occurred rarely when the tumor margin could not be clearly defined during surgery.

### Statistical analysis

For intergroup comparison, the Student t test was used for parametric data and the Mann-Whitney U test for nonparametric data. Percentages were compared via the chi-square test or the Fisher exact test. In univariate analysis, variables with p<0.2 entried into subsequent multivariate logistic regression analysis to determine the more important risk factors. Differences were considered significant with *p* < 0.05. The results are presented as odds ratio (OR) and 95% confidence intervals. Analyses were performed using IBM SPSS Statistics 20 (IBM Corp., Armonk, New York, USA).

## Results

### Patient characteristics

Two hundred and seventy-two patients underwent intracanal tumors resection were reviewed in this study. Demographic, clinical, and surgical features are summarized in Table 1. One hundred and twenty-seven (46.7%) patients were male, 145 (53.3%) patients were female, and their average age was 42.56 ± 16.18 years at the time of surgery. The average BMI index was 23.28 ± 3.49. One hundred and fifty-two patients (55.9%) presented with back pain symptoms, 139(51.1%) with motor weakness symptoms, 108(39.7%) with sensory abnormal symptoms, and 47(17.3%)with sphincter disturbances. The average symptom duration was 19.65(0–240) months. The median preoperative MMS score was 3 (interquartile range [IQR] 2–3). The number of patients with tumors located in the cervical spine, thoracic spine, lumbar spine, the cervicothoracic junction (C-7 and/or T-1), and the thoracolumbar junction (T-12 and/or L-1) were 50 (18.4%), 103 (37.9%), 70 (25.7%), 20 (7.4%), and 29 (10.7%) respectively. Four (1.5%) patients underwent preoperative biopsy. One patient underwent chemotherapy before surgery.

**Table 1:** Baseline Patient Demographics, Comorbidities, and Operative Factors

Variable	Value
Sex, n(%)	
Female	145 (53.3%)
Male	127 (46.7%)
Age in yrs	42.56 ± 16.18
BMI (kg/m^2^)	23.28 ± 3.49
Symptom duration in mos	19.65 (0–240)
Presenting symptoms	
Back pain, n(%)	152 (55.9%)
Motor weakness, n (%)	139 (51.1%)
Sensory abnormal, n (%)	108 (39.7%)
Sphincter disturbances, n (%)	47 (17.3%)
Median preop MMS score (IQR)	3 (2–3)
Location, n (%)	
Cervical	50 (18.4%)
Cervicothoracic	20 (7.4%)
Thoracic	103 (37.9%)
Thoracolumbar	29 (10.7%)
Lumbar	70 (25.7%)
Vertebral levels of tumor involvement, n (%)	
1	69 (25.4%)
2	136 (50%)
3	39 (14.3%)
4	15 (5.5%)
5	3 (1.1%)
6	2 (0.7%)
7	4 (1.5%)
8	1 (0.4%)
12	3 (1.1%)
Previous treatment, n(%)	
Biopsy	4 (1.5%)
Chemotherapy	1 (0.4%)
Preop spinal deformity, n(%)	
No	275 (97.4%)
Yes	7 (2.6%)
Postop spinal deformity, n(%)	
No	229 (84.2)
Yes	43 (15.8)

### Surgical and tumor characteristics

The extent of resection of the laminae was depended on the vertebral levels of tumor involvement. Generally, keep the lamina as much as possible during the resection. Two hundred and fifty-three (93%) underwent laminoplasty and 19 (7%) underwent laminectomy. Gross total resection was achieved in 255 patients (93.8%). Subtotal resection was achieved in 115 (5.5%). Partial resection was achieved in 2(0.7%). Pathology was intradural in 234 (86%) and extradural in 38(14%). Pathology included neurinoma in 123 (45.2%), meningioma in 41 (15.1%), ependymoma in 23 (8.5%), cyst in 15 (5.5%), angioma in 19 (7%), and others in 51 (18.8%) (Table 2).

**Table 2:** Surgical and tumor characteristics

Sugery methods (laminectomy or laminoplasty)	
laminoplasty	253 (93%)
laminectomy	19 (7%)
Extent of Surgery resection (no. of spinal levels)	
1	53 (19.5%)
2	151 (55.5%)
3	44 (16.2%)
4	16 (5.9%)
5	3 (1.1%)
7	3 (1.1%)
8	1 (0.4%)
10	1 (0.4%)
Extent of tumor resection	
GTR	255 (93.8%)
STR	15 (5.5%)
Partial resection	2 (0.7%)
Intramedullary or Extramedullary, n(%)	
Intramedullary	45 (16.5%)
Extramedullary	237 (83.5%)
Surgeon, n (%)	
Dr. Lin	98 (36%)
Dr. Meng	50 (18.4%)
Dr. Cui	38 (14%)
Dr. Gao	36 (13.2%)
Dr. Wu	25 (9.2%)
Other	25 (9.2%)
Pathology, n (%)	
Neurinoma	123 (45.2%)
Meningioma	41 (15.1%)
Ependymoma	23 (8.5%)
Cyst	15 (5.5%)
Angioma	19 (7%)
Other	51 (18.8%)

### Postoperative outcomes and complications

Surgical site infection occurred in 7 patients (2.6%). Incisional cerebrospinal fluid (CSF) leak occurred in 9(3.3%). Neurologic complications, such as the emerging sphincter disturbances, sensory abnormal symptoms and even paraplegia, during hospitalization occurred in 19(7%). The mean postoperative length of hospital stay was 16.79 ± 8.34 days. The average follow-up time was 21.8(6–114) mouths. Two hundred fifty-six people underwent postoperative brace fixation. The median postoperative MMS score at last follow-up was 1 (interquartile range [IQR] 1–1) (Table 3).

**Table 3:** Postoperative outcomes and complications

Surgical site infection, n (%)	7 (2.6%)
Incisional CSF leak, n (%)	9 (3.3%)
Neurologic complications during hospitalization, n (%)	19 (7%)
Postoperative length of hospital stay, n	16.79 ± 8.34
Median MMS score at last FU (IQR)	1 (1–1)
Mean FU in mos (range)	21.82 (6–114)
Time of spinal deformity in mos (range)	13.23 (2–60)
Postoperative brace fixation, n(%)	256 (94.1%)

### Incidence of progressive spinal deformity

**Fig. 2:**
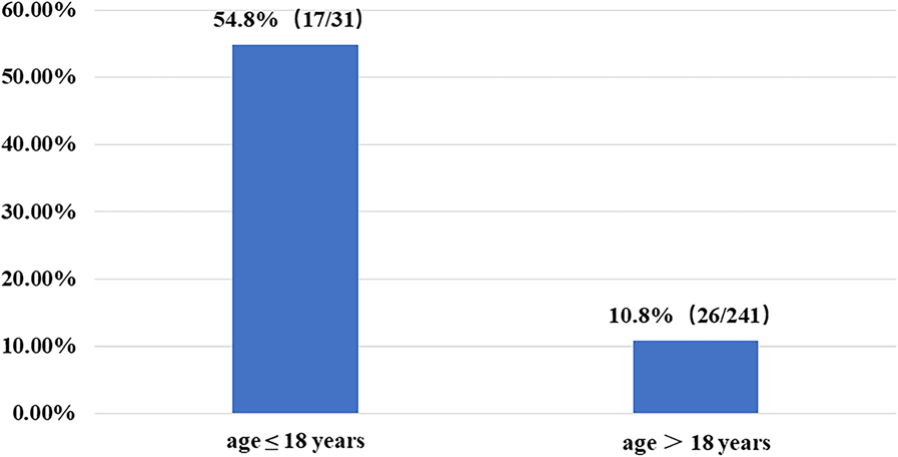
Age distribution of progressive spinal deformity in patients who underwent resection of intracanal tumors

Forty-three (15.8%) patients developed progressive radiographic deformity within a mean of 13.2 months after surgery. Among them, there were 26 adult (> 18 years old) patients, accounting for 10.8% of all 241 adult patients, and 17 children (≤18 years of age), accounting for 54.8% of the 31 pediatric patients (Fig. 2). Among these 43 patients, 30 developed progressive kyphosis, and 13 developed progressive lordosis. Meanwhile, 7 patients presented with preoperative spinal deformity, and the extent of spinal deformity were deteriorated after surgery in all of them (Table 4). Four (9.3%) patients with radiographic spinal deformity had symptoms and the others did not. Two (4.7%) patients underwent spinal fusion surgery (Table 4).

**Table 4:** Clinical features of patients who developed progressive spinal deformity following SCTs resection

Case (#)	Sex	Age range (yrs)	Spinal segment (C/T/L/S)	TumorlocatIon (I/E)	Preop Spinal Deformity (yes/no)	Types of preoperative spinal deformity (yes/no)	Sugery methods	Number of levels	Surgeon	Types of postoperative spinal deformity	Time of spinal deformity in mos	Symptoms of occuranceof radiological deformity (yes/no)	Pathology	Grade	Extent of tumor resection	ClinicalStatus	Postoperative brace fixation (yes/no)	Postoperative spinal fusion (yes/no)	Pre-op MMS	MMS at FU	CSF leak	FU (mos)	
1	M	31–35	L	E	no	no	Laminoplasty	3	Dr. Lin	kyphosis	10	no	Cyst	low	GTR	Improved	yes	no	4	2	No	10	
2	M	81–85	L	I	no	no	Laminoplasty	1	Dr. Lin	lordosis	32	no	Neurinoma	low	GTR	Improved	yes	no	3	1	No	32	
3	F	51–55	C	I	no	no	Laminoplasty	4	Dr. Gao	kyphosis	3	no	Neurinoma	low	GTR	Improved	yes	no	3	1	No	7	
4	M	51–55	T	I	no	no	Laminoplasty	2	Dr. Cui	kyphosis	32	no	Meningioma	low	GTR	Improved	yes	no	2	1	No	32	
5	M	46–50	L	I	no	no	Laminoplasty	1	Dr. Wu	lordosis	3	no	Neurinoma	low	GTR	Improved	yes	no	2	1	No	7	
6	M	11–15	T	I	yes	Scoliosis	Laminoplasty	2	Dr. Lin	Scoliosis; kyphosis	29	yes	Neurinoma	low	GTR	Improved	no	no	2	1	No	52	
7	F	6–10	CT	I	no	no	Laminoplasty	7	Dr. Lin	kyphosis	4	no	Ependymoma	High	GTR	Improved	yes	no	3	1	No	6	
8	M	11–15	T	I	yes	Scoliosis	Laminoplasty	2	Dr. Lin	Scoliosis; kyphosis	6	no	Neurinoma	low	GTR	Improved	no	no	2	1	no	48	
9	M	6–10	T	I	no	no	Laminoplasty	3	Dr. Wu	kyphosis	3	no	Neurinoma	low	GTR	Improved	yes	no	4	2	no	3	
10	M	16–20	C	I	yes	Scoliosis	Laminoplasty	2	Dr. Lin	Scoliosis; lordosis	6	no	Neurinoma	low	GTR	Improved	yes	no	2	1	no	7	
11	F	1–5	L	I	no	no	Laminoplasty	2	Dr. Cui	lordosis	5	no	Other	high	GTR	Improved	yes	no	3	1	no	6	
12	F	11–15	T	I	no	no	Laminoplasty	2	Dr. Lin	kyphosis	3	yes	Other	low	GTR	improved	yes	no	3	2	no	11	
13	M	11–15	CT	I	no	no	Laminoplasty	3	Dr. Lin	kyphosis	4	no	glioma	high	Partial resection	Improved	yes	no	3	1	no	21	
14	F	41–45	T	I	no	no	Laminoplasty	2	Dr. Lin	kyphosis	14	no	Ependymoma	high	GTR	improved	yes	no	3	1	no	14	
15	F	26–30	CT	I	no	no	Laminoplasty	10	Dr. Lin	kyphosis	2	no	Ependymoma	high	GTR	improved	yes	no	3	1	no	25	
16	M	16–20	C	E	no	no	Laminoplasty	2	Other	kyphosis	27	no	Neurinoma	low	GTR	improved	yes	no	3	1	no	39	
17	M	26–30	T	I	yes	Scoliosis	Laminoplasty	3	Dr. Lin	Scoliosis; kyphosis	6	no	Other	high	GTR	Improved	yes	no	3	1	no	6	
18	F	41–45	L	I	no	no	Laminoplasty	2	Dr. Cui	lordosis	15	no	Neurinoma	low	GTR	Improved	yes	no	3	1	no	27	
19	F	21–25	C	E	no	no	Laminoplasty	3	Dr. Wu	kyphosis	39	no	Cyst	low	GTR	Improved	yes	no	2	1	no	39	
20	M	11–15	L	I	no	no	Laminoplasty	7	Dr. Lin	lordosis	2	no	Immature teratoma	high	GTR	Improved	no	no	5	4	no	3	
21	F	41–45	T	I	no	no	Laminoplasty	3	Dr. Meng	kyphosis	26	yes	Meningioma	low	GTR	Worsen	yes	no	3	4	no	28	
22	M	6–10	TL	I	yes	kyphosis	Laminoplasty	2	Dr. Lin	kyphosis	3	yes	Cyst	low	GTR	Improved	yes	yes	2	1	no	24	
23	F	46–50	L	I	no	no	Laminoplasty	1	Dr. Lin	lordosis	14	no	Neurinoma	low	GTR	Improved	yes	no	3	1	no	14	
24	F	11–15	TL	I	no	Scoliosis	Laminoplasty	2	Dr. Lin	Scoliosis; lordosis	60	no	Cyst	low	GTR	Improved	yes	yes	2	1	no	60	
25	F	61–65	C	I	no	no	Laminoplasty	3	Other	kyphosis	2	no	Meningioma	low	GTR	Improved	yes	no	3	1	No	2	
26	M	41–15	L	I	no	no	Laminoplasty	2	Other	lordosis	6	no	Cyst	low	GTR	Improved	yes	no	3	1	no	6	
27	F	41–45	TL	I	no	no	Laminoplasty	3	Dr. Cui	kyphosis	3	no	Cyst	low	GTR	Improved	yes	no	3	1	no	3	
28	M	11–15	CT	I	no	no	Laminoplasty	2	Dr. Meng	kyphosis	11	no	Neurinoma	low	GTR	Improved	yes	no	3	1	no	39	
29	M	6–10	TL	I	no	no	Laminoplasty	2	Dr. Cui	kyphosis	6	no	Ependymoma	High	STR	Improved	yes	no	4	1	no	54	
30	M	31–35	T	I	no	no	Laminoplasty	4	Dr. Lin	kyphosis	60	no	glioma	high	GTR	Improved	yes	no	3	1	no	60	
31	M	16–20	LS	I	no	no	Laminoplasty	1	Dr. Wu	lordosis	3	no	Neurinoma	low	GTR	Improved	yes	no	2	1	no	3	
32	F	11–15	T	I	yes	Scoliosis	Laminoplasty	8	Dr. Lin	Scoliosis; kyphosis	60	no	Other	low	GTR	Improved	yes	no	3	1	no	60	
33	F	41–45	L	I	no	no	Laminoplasty	2	Dr. Cui	lordosis	3	no	Neurinoma	low	GTR	Improved	yes	no	2	1	No	18	
34	M	41–45	CT	I	no	no	Laminoplasty	2	Dr. Lin	kyphosis	3	no	Ependymoma	high	GTR	Improved	yes	no	2	1	no	19	
35	F	41–45	T	I	no	no	Laminoplasty	2	Dr. Cui	kyphosis	3	no	Neurinoma	low	GTR	Improved	yes	no	2	1	no	36	
36	M	11–15	TL	I	no	no	Laminoplasty	2	Dr. Cui	kyphosis	10	no	Neurinoma	low	GTR	Improved	yes	no	2	1	no	10	
37	F	31–35	T	I	no	no	Laminoplasty	1	Dr. Lin	kyphosis	3	no	Angioma	low	GTR	Improved	yes	no	3	1	No	14	
38	M	46–50	C	I	no	no	laminectomy	2	Dr. Cui	kyphosis	3	no	Other	low	GTR	Improved	yes	no	3	1	no	6	
39	M	46–50	C	I	no	no	laminectomy	2	Dr. Meng	kyphosis	6	no	Neurinoma	low	GTR	Improved	yes	no	3	1	no	4	
40	F	41–45	T	I	no	no	laminectomy	2	Dr. Lin	kyphosis	5	no	Meningioma	low	GTR	Improved	yes	no	2	1	no	5	
41	M	46–50	T	I	no	no	laminectomy	2	Dr. Wu	kyphosis	29	no	Neurinoma	low	GTR	Improved	yes	no	3	2	no	29	
42	M	11–15	CT	I	no	no	laminectomy	3	Dr. Gao	lordosis	2	no	Immature teratoma	High	GTR	Improved	yes	no	5	3	yes	1	
43	F	41–45	C	I	no	no	Laminoplasty	2	Other	lordosis	3	no	Meningioma	High	GTR	Improved	yes	no	3	1	yes	6	

### Risk factors for progressive spinal deformity

In the univariate analysis, age (*p* = 0.000),sex(*p* = 0.191), BMI(*p* = 0.000), symptom duration in mouths(*p* = 0.000), median preop MMS score(*p* = 0.019), location of tumor (*p* = 0.151), vertebral levels of tumor involvement (*p* = 0.005), preoperative biopsy(*p* = 0.013), preoperative spinal deformity (*p* = 0.000), extent of surgery resection involvement (*p* = 0.000), surgeon(*p* = 0.078), pathology(*p* = 0.085), median MMS score at last follow-up (*p* = 0.114), and intramedullary or not(*p* = 0.082) with a *P* value < 0.2 were identified as factors associated with postoperative progressive spinal deformity (Table 5). In subsequent multivariate logistic regression analysis, age < 18 years (*p* = 0.027), vertebral levels of tumor involvement (*p* = 0.019) and preoperative spinal deformity(*p* = 0.008) were the independent risk factors (*p* < 0.05), increasing the odds of postoperative progressive spinal deformity by 3.94-, 0.69- and 27.11-fold, respectively (Table 6).

**Table 5:** Univariate analysis for predicting risk factors of progressive spinal deformity

Variable	*p* value
Sex	**0.191**
Age	**0.000**
BMI	**0.000**
Symptom duration in mos	**0.000**
Presenting symptoms	
Back pain	0.497
Motor weakness	0.181
Sensory abnormal	0.166
Sphincter disturbances	0.850
Median preop MMS score	**0.019**
Location of tumor	**0.151**
Vertebral levels of tumor involvement	**0.005**
Previous treatment	
Biopsy	**0.013**
Chemotherapy	1.000
Preop spinal deformity	**0.000**
Extent of Surgery resection involvement	**0.000**
Extent of tumor resection	0.750
Intramedullary or Extramedullary	**0.082**
Surgeon	**0.078**
Pathology	**0.085**
Median MMS score at last FU	**0.114**

Data set in bold are statistically significant

**Table 6:** Multivariate logistic regression analysis for predicting risk factors of progressive spinal deformity in patients who underwent laminoplasty or laminectomy

Variable	OR	95% CI	*p* value
Sex	0.812	0.325–2.033	0.657
Age ≤ 18 years	3.941	1.165–13.327	**0.027**
BMI	1.105	0.971–1.256	0.129
Symptom duration in mos	1.006	0.99–1.022	0.497
Median preop MMS score	0.700	0.385–1.275	0.244
Location of tumor			0.413
Cervical	2.628	0.712–9.706	0.147
Cervicothoracic	0.743	0.164–3.358	0.699
Thoracic	1.650	0.526–5.175	0.39
Thoracolumbar	2.436	0.527–11.263	0.255
Vertebral levels of tumor involvement	0.697	0.516–0.942	**0.019**
Previous treatment	2.703	0.121–60.35	0.53
Preop spinal deformity	27.112	2.408–305.316	**0.008**
Extent of Surgery resection involvement	1.061	0.691–1.63	0.785
Surgeon			0.226
Dr. Lin	0.739	0.168–3.247	0.688
Dr. Meng	2.490	0.393–15.763	0.332
Dr. Cui	0.472	0.102–2.19	0.338
Dr. Gao	2.844	0.404–20.005	0.294
Dr. Wu	0.802	0.144–4.46	0.801
Pathology			0.634
Neurinoma	0.443	0.14–1.407	0.167
Meningioma	0.375	0.087–1.612	0.187
Ependymoma	0.478	0.104–2.2	0.343
Cyst	0.378	0.074–1.948	0.245
Angioma	1.156	0.121–11.085	0.9
Median MMS score at last FU	0.852	0.403–1.802	0.674
Intramedullary or Extramedullary	0.497	0.188–1.312	0.158

Data set in bold are statistically significant

## Discussion

Postoperative progressive spinal deformity has been reported as an important complication following intracanal tumors resection. Deformity may develop progressively within many years after surgery and affect the final outcomes of patients [7]. However, opinions varied about the risk factors for postoperative progressive spinal deformity [5, 6]. Since first described in 1976 [13], laminoplasty has gradually replaced laminectomy for the less damages to the structure of the vertebral body and lower incidence of postoperative complications, such as incisional CSF leak [10]. However, some studies reported that laminoplasty was not associated with improvement in postoperative deformity after tumor resection [8, 10]. Here, we analyzed the risk factors for postoperative spinal deformity following intracanal tumors resection, hoping to arouse the attention of the surgeons to reduce the occurrence of such complication. For those patients who had more risk factors of progressive spinal deformity, spinal fusion surgery may be seriously considered, and close follow-up should be given to those who did not undergo this procedure.

In this research, 272 patients with resection of intracanal tumors were presented and risk factors of progressive spinal deformity were evaluated. After an average of 21.8 months of follow-up, the overall incidence of postoperative progressive spinal deformity was 15.8%, which was comparable to previously reported incidence. We included the current most factors to analyze. Our research revealed that age ≤ 18 years (*p* = 0.027), vertebral levels of tumor involvement (*p* = 0.019) and preoperative spinal deformity (*p* = 0.008) was the independent risk factors (*p* < 0.05), increasing the odds of postoperative progressive spinal deformity by 3.94-, 0.69- and 27.11-fold, respectively. Meanwhile, the patients with progressive spinal deformity had a trend of increased postoperative median MMS score at last follow-up (*p* = 0.199) and neurologic complications. To date, this is the largest reported risk factor analysis case series in this field. Not only does it contain the largest number of cases, but also the factors. Moreover, it involved intramedullary and extramedullary tumors.

Papagelopoulos et al. [11, 14] reported that the incidence of spinal column deformity was 33% in children and adolescents while 8% in young adults. Recently, Wei Shi et al. [11] reported that patient age ≤ 25 was the main significant predictive risk factor for postoperative spinal deformity. These data were consistent with our results that pediatric patients (≤ 18 years of age) were more likely to suffer from postoperative progressive spinal deformity than the older adults (> 18 years of age). We speculated that the pediatric patient’s immature skeletal system as well as surgery itself may change the mechanics of the spine, contributing to this phenomenon. In addition, because the growth rate of bone growth in children was greater than the spinal cord, adhesions in the postoperative area may cause a phenomenon similar with tethered cord syndrome, which caused related muscle neurotrophic decline that contributed to the development of spinal deformity.

Many previous studies demonstrated that extent of surgery resection (no. of spinal levels) was related to progressive spinal deformity. Katsumi et al. [15] revealed that age at operation, preoperative curvature in neutral position, number of removed laminas, C2 laminectomy, and destruction of facet joints are the risk factors that are involved in the pathogenetic mechanism of cervical instability. However, in our research, our data showed that not the extent of surgery resection, but the vertebral levels of tumor involvement may cause instability of the spine. The study revealed the average level of tumor involvement was 3.4 in patients who had progressive spinal deformity, comparing with 2 who had not. The risk factor of the vertebral levels of tumor involvement increased the odds of postoperative progressive spinal deformity by 0.69- fold. The more levels of tumor involvement, the more severe compression of the spinal cord, which may led to neurotrophic decline that aggravated the occurrence of spinal deformity.

Preoperative spinal deformity was independently associated with development of postoperative spinal deformity [16]. Similarly, Kaptain GJ et al. [11, 17] reported that the presence of preoperative spinal deformity was the factor most significantly related to the risk of developing progressive spinal deformity. Our research got the same conclusion. Preoperative spinal deformity even increased the odds of postoperative progressive spinal deformity by 27.11-fold, and it was the biggest risk factor in the occurrence of postoperative spinal deformity. Preoperative spinal deformity may partly result from the spinal cord compression by the tumors, especially when the tumors invaded into the anterior horn region, which in turn led to neurotrophic disorders of the paravertebral muscles in the corresponding segments. The imbalance of paravertebral muscle caused a decrease in the stability of the spine. Eventually it led to progressive deformity. The operation inevitably damaged the posterior ligamentous complex and paraspinal muscles, which would further aggravate the preoperative spinal instability.

Riseborough et al. [18] reported that the greater amount of irradiation could lead to more severe deformity of the spine. Although the prior radiotherapy was not analyzed because of few relevant cases in our study, we found the patients with spinal deformity were more likely to suffer from preoperative puncture or biopsy, which might destroy the stability of the spine to some extent. Moreover, in the univariate analysis, extent of surgery resection involvement was also significantly higher in patients with spinal deformity than that without spinal deformity. However, in subsequent multivariate analysis, we found that compared with younger age and preoperative spinal deformity, the extent of surgery resection involvement contributed little to postoperative progressive spinal deformity. Many previous studies revealed that number of laminae resected played a role in the development of postoperative spinal deformity [6]. Here, we recommended that when encountering the tumors involved in multiple segments, under the premise of ensuring complete resection, minimize the number of laminae resected to minimize the loss of spinal stability.

Furthermore, the pathology of the tumors, location of tumor and the surgeons may influence the development of postoperative spinal deformity [16, 19]. Our research also showed this trend (pathology, *p* = 0.085; surgeon, *p* = 0.078), but they did not reach statistical significance. In addition, our study showed that methods (laminectomy or laminoplasty, *p* = 0.746) didn’t affect the occurrence of postoperative progressive spinal deformity.

## Conclusions

We found that the patients who had younger age (< 18 years), tumors involved in multiple segments and preoperative spinal deformity had more risks of having postoperative progressive spinal deformity. The risk factors of postoperative progressive spinal deformity warrant serious reconsideration that when performing resection of intracanal tumors in these patients with more risk factors, the surgeons should seriously consider to conduct follow-up more closely or provide surgical fusion in order to reduce the risk of reoperation and improve the prognosis of patients.

## Data Availability

All the medical records of the patients underwent SCTs resection between January 2009 and December 2018 are from the medical record system of Chongqing Southwest Hospital. We organized and categorized the data to make it easier to access and analyze. If anyone would like to access to the original data, please contact us.

## Abbreviations

BMI: Body Mass Index
CSF: Cerebrospinal fluid
CT: Computed Tomography
IQR: Interquartile range
MMS: McCormick scale
MR images: Magnetic Resonance Images
MRI: Magnetic Resonance Imaging
OR: Odds ratio
SCTs: Spinal cord tumors
